# Supplementary material for: Integrative visual omics of the white-rot fungus Polyporus brumalis exposes the biotechnological potential of its oxidative enzymes for delignifying raw plant biomass
Source: Biotechnol Biofuels. 2018 Jul 23;11:201. doi: 10.1186/s13068-018-1198-5 (PMC6055342; doi:10.1186/s13068-018-1198-5)
Supplement: Supplementary file 1 — Additional file 1: Table S1. List of predicted Auxiliary Activity enzymes from CAZy families AA2 and AA3 encoded in the genome of Polyporus brumalis BRFM 1820. Expert annotations for AA3 sub-families, versatile peroxidases (VP), manganese peroxidases (MnP) and generic peroxidases (GP) are indicated. The Cellobiose Dehydrogenase with the modular structure AA8-AA3_1, ProtID #1364243, is not indicated. [file 13068_2018_1198_MOESM1_ESM.docx]

**Table S1.** List of predicted Auxiliary Activity enzymes from CAZy families AA2 and AA3 encoded in the genome of *Polyporus brumalis* BRFM 1820. Expert annotations for AA3 sub-families, Versatile Peroxidases (VP), Manganese Peroxidases (MnP) and Generic Peroxidases (GP) are indicated. The Cellobiose Dehydrogenase with the modular structure AA8-AA3_1, ProtID #1364243, is not indicated.

| ProtID | CAZyme family | predicted secreted |
| --- | --- | --- |
| 1404761 | AA3_4 | no |
| 1364967 | AA3_3 | no |
| 1467319 | AA3_3 | no |
| 1484504 | AA3_3 | no |
| 823803 | AA3_2 | yes |
| 1051827 | AA3_2 | no |
| 1327691 | AA3_2 | Fragment N-term |
| 1343556 | AA3_2 | yes |
| 1349632 | AA3_2 | no |
| 1349968 | AA3_2 | yes |
| 1352226 | AA3_2 | yes |
| 1358588 | AA3_2 | yes |
| 1388653 | AA3_2 | no |
| 1394538 | AA3_2 | yes |
| 1399257 | AA3_2 | yes |
| 1399288 | AA3_2 | yes |
| 1407962 | AA3_2 | yes |
| 1408514 | AA3_2 | no |
| 1410843 | AA3_2 | no |
| 1410997 | AA3 | no |
| 1414090 | AA3_2 | no |
| 1422328 | AA3_2 | no |
| 1424706 | AA3_2 | no |
| 1455149 | AA3_2 | yes |
| 1458629 | AA3_2 | no |
| 1477130 | AA3_2 | no |
| 1479630 | AA3_2 | no |
| 1481505 | AA3 | no |
| 1483588 | AA3_2 | no |
| 1483602 | AA3_2 | no |
| 1488846 | AA3_2 | no |
| 1490162 | AA3_2 | yes |
| 1509436 | AA3_2 | yes |
| 1519516 | AA3_2 | no |
| 1519533 | AA3_2 | no |
| 1538961 | AA3_2 | no |
| 310665 | AA2 (VP) | yes |
| 855582 | AA2 (MnP-short) | yes |
| 897918 | AA2 (VP) | yes |
| 918032 | AA2 (VP) | yes |
| 935251 | AA2 (VP) | yes |
| 1110945 | AA2 (VP) | yes |
| 1185543 | AA2 (MnP-short) | yes |
| 1340324 | AA2 (MnP-short) | yes |
| 1347226 | AA2 (MnP-short) | yes |
| 1359988 | AA2 (VP) | yes |
| 1360396 | AA2 (VP) | yes |
| 1363610 | AA2 (MnP-short) | yes |
| 1412926 | AA2 (VP) | yes |
| 1418086 | AA2 (MnP-short) | yes |
| 1486819 | AA2 (MnP-short) | yes |
| 1487275 | AA2 (VP) | yes |
| 1487289 | AA2 (MnP-short) | Fragment C-term |
| 1487292 | AA2 (MnP-short) | Fragment C-term |
| 1560842 | AA2 (GP) | Fragment N-term |
