# Supplementary material for: Integrative visual omics of the white-rot fungus Polyporus brumalis exposes the biotechnological potential of its oxidative enzymes for delignifying raw plant biomass
Source: Biotechnol Biofuels. 2018 Jul 23;11:201. doi: 10.1186/s13068-018-1198-5 (PMC6055342; doi:10.1186/s13068-018-1198-5)

**Figure S1.** Distributions of the normalized log2 transformed read count of all biological replicates. **A**: Box plot showing the mean and the range of the read count of the genes. **B**: Density plot showing distributions of log2 read count of the genes. **Liq**: Liquid cultivation on malt extract for 10 days. **Day4**: Solid-state cultivation on wheat straw for 4 days after liquid cultivation on malt extract for 6 days. **Day10**: Solid-state cultivation on wheat straw for 10 days after liquid cultivation on malt extract for 6 days. **Day15**: Solid-state cultivation on wheat straw for 15 days after liquid cultivation on malt extract for 6 days.

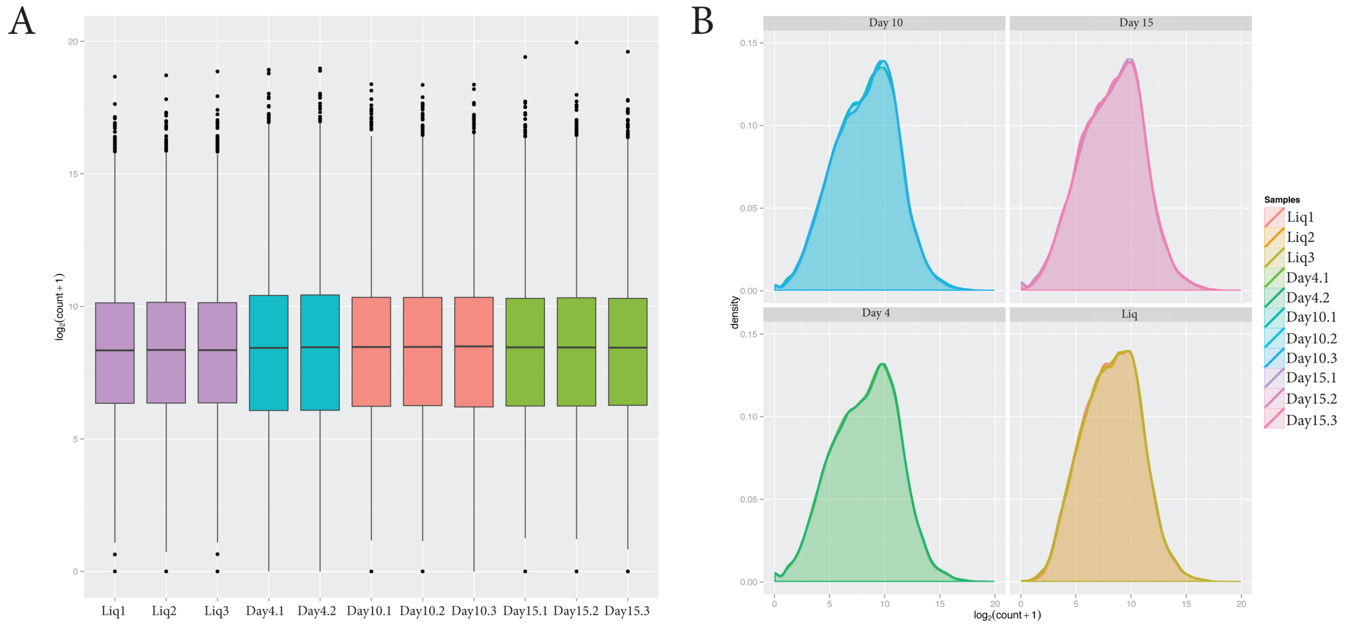

Supplement: Supplementary file 2 — Additional file 2: Figure S1. Distributions of the normalized log2 transformed read count of all biological replicates. Liq: Liquid cultivation on malt extract for 10 days. Day4: Solid-state cultivation on wheat straw for 4 days after liquid cultivation on malt extract for 6 days. Day10: Solid-state cultivation on wheat straw for 10 days after liquid cultivation on malt extract for 6 days. Day15: Solid-state cultivation on wheat straw for 15 days after liquid cultivation on malt extract for 6 days. A: Box plot showing the mean and the range of the read count of the genes. B: Density plot showing distributions of log2 read count of the genes. [file 13068_2018_1198_MOESM2_ESM.pdf]
