# Supplementary material for: Integrative visual omics of the white-rot fungus Polyporus brumalis exposes the biotechnological potential of its oxidative enzymes for delignifying raw plant biomass
Source: Biotechnol Biofuels. 2018 Jul 23;11:201. doi: 10.1186/s13068-018-1198-5 (PMC6055342; doi:10.1186/s13068-018-1198-5)
Supplement: Supplementary file 3 — Additional file 3: Table S2. The number of highly transcribed genes in the selected nodes (> mean 12 log2 normalized read count per node) in response to the solid and liquid conditions. Total: The total number of genes including the unique and shared genes. Unique: specifically highly transcribed in each condition. Shared: Highly transcribed in both conditions. Node IDs containing such genes are provided (Additional file 7: Table S4). [file 13068_2018_1198_MOESM3_ESM.docx]

**Table S2.** The number of highly transcribed genes in the selected nodes (> mean 12 log2 normalized read count per node) in response to the solid and liquid conditions. **Total**: The total number of genes including the unique and shared genes. **Unique**: Specifically highly transcribed in each condition. **Shared**: Highly transcribed in both conditions. Node IDs containing such genes are provided (Table S4).

| Condition | Total | Unique | Shared |
| --- | --- | --- | --- |
| Solid | 1525 | 727 | 798 |
| Liquid | 1076 | 278 |  |
