# Supplementary material for: Integrative visual omics of the white-rot fungus Polyporus brumalis exposes the biotechnological potential of its oxidative enzymes for delignifying raw plant biomass
Source: Biotechnol Biofuels. 2018 Jul 23;11:201. doi: 10.1186/s13068-018-1198-5 (PMC6055342; doi:10.1186/s13068-018-1198-5)
Supplement: Supplementary file 5 — Additional file 5: Figure S2. KOG classification of the genes differentially highly transcribed after 4-day growth in SSF on wheat straw as compared to control liquid cultures. The identified genes were clustered into nodes of co-regulated genes with mean Log2 of the normalized read counts > 12 in SSF and < 12 in control liquid cultures. The number of up-regulated genes in each KOG class is indicated for the KOG groups Cellular Processes and Signaling, Information storage and Processing and Metabolism. [file 13068_2018_1198_MOESM5_ESM.pdf]

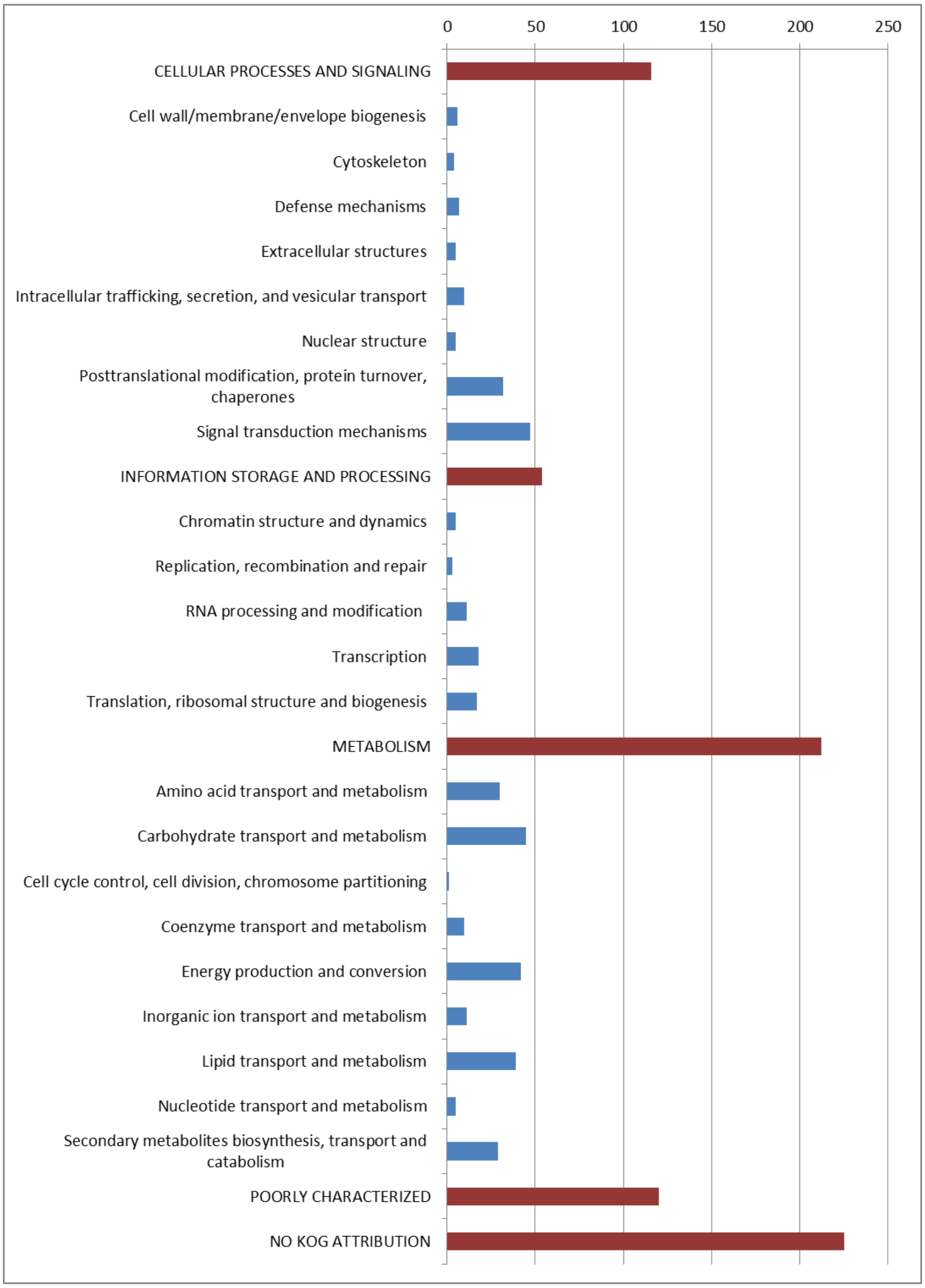

Figure S2. KOG classification of the genes differentially highly transcribed after 4-day growth in SSF on wheat straw as compared to control liquid cultures. The identified genes were clustered into nodes of co-regulated genes with mean log2 of the normalized read counts >12 in SSF and <12 in control liquid cultures. The number of up-regulated genes in each KOG class is indicated for the KOG groups Cellular Processes and Signaling, Information storage and Processing and Metabolism.
