# Supplementary material for: Integrative visual omics of the white-rot fungus Polyporus brumalis exposes the biotechnological potential of its oxidative enzymes for delignifying raw plant biomass
Source: Biotechnol Biofuels. 2018 Jul 23;11:201. doi: 10.1186/s13068-018-1198-5 (PMC6055342; doi:10.1186/s13068-018-1198-5)
Supplement: Supplementary file 6 — Additional file 6: Table S3. CAZyme coding genes present in nodes with mean log2 read counts < 12 in control liquid cultures and mean log2 read counts > 12 at Day 4 in SSF. [file 13068_2018_1198_MOESM6_ESM.docx]

**Table S3.** CAZyme coding genes present in nodes with mean log2 read counts <12 in control liquid cultures and mean log2 read counts >12 at Day 4 in SSF.

| **protID** | **nodeID** | **detected in the secretome at Day 4** | **control log2 read counts** | **Day 4 log2 read counts** | **CAZyme family** |
| --- | --- | --- | --- | --- | --- |
| 1486819 | 372 | yes | 8,52 | 16,89 | AA2 MnP-short |
| 1359988 | 400 | yes | 6,73 | 17,36 | AA2 VP |
| 1487275 | 417 | yes | 9,84 | 18,95 | AA2 VP |
| 897918 | 418 | yes | 8,06 | 15,10 | AA2 VP |
| 1412926 | 418 | yes | 7,42 | 14,95 | AA2 VP |
| 1360396 | 419 | yes | 6,40 | 18,95 | AA2 VP |
| 918032 | 419 | yes | 7,31 | 17,99 | AA2 VP |
| 1185543 | 420 | yes | 7,00 | 12,95 | AA2 MnP-short |
| 1347226 | 420 | yes | 6,63 | 12,72 | AA2 MnP-short |
| 1487292 | 422 | no | 5,49 | 15,94 | AA2 MnP-short |
| 1399288 | 209 | no | 11,43 | 13,63 | AA3_2 |
| 1364967 | 417 | no | 10,76 | 17,12 | AA3_3 |
| 713930 | 418 | no | 8,38 | 17,61 | AA5 |
| 1557562 | 418 | yes | 6,06 | 16,87 | AA5 |
| 1420749 | 7 | yes | 6,26 | 14,31 | AA9 |
| 1452362 | 26 | yes | 7,62 | 14,89 | AA9 |
| 1456024 | 31 | yes | 7,43 | 13,03 | AA9 |
| 1403153 | 47 | yes | 8,87 | 13,72 | AA9 |
| 1339229 | 372 | yes | 7,41 | 15,00 | AA9 |
| 1456088 | 2 | no | 7,04 | 13,93 | CE1 |
| 139112 | 26 | yes | 8,44 | 14,67 | CE1 |
| 832761 | 420 | yes | 5,89 | 13,41 | CE12 |
| 1486135 | 2 | yes | 5,39 | 14,03 | CE15 |
| 1404492 | 2 | yes | 7,67 | 13,56 | CE16 |
| 1395444 | 397 | no | 8,47 | 11,36 | CE16 |
| 1412498 | 420 | no | 6,99 | 12,54 | CE16 |
| 1520578 | 25 | no | 8,24 | 11,88 | GH10 |
| 659690 | 31 | yes | 7,78 | 14,75 | GH10 |
| 16190 | 397 | no | 7,95 | 12,55 | GH10 |
| 1486161 | 421 | no | 4,55 | 14,10 | GH10 |
| 1351738 | 27 | yes | 6,42 | 12,58 | GH12 |
| 492772 | 29 | yes | 7,84 | 13,41 | GH131 |
| 1349191 | 47 | yes | 9,56 | 13,41 | GH15 |
| 1484581 | 209 | yes | 11,18 | 13,28 | GH16 |
| 1351991 | 3 | yes | 9,20 | 13,10 | GH18 |
| 1437352 | 397 | no | 7,84 | 11,58 | GH2 |
| 1498325 | 395 | no | 9,81 | 12,50 | GH27 |
| 1447784 | 2 | yes | 7,62 | 14,62 | GH28 |
| 593055 | 7 | yes | 7,10 | 13,00 | GH3 |
| 1364811 | 27 | no | 6,31 | 12,36 | GH3 |
| 224000 | 209 | no | 10,53 | 12,90 | GH3 |
| 615361 | 209 | no | 10,86 | 12,82 | GH3 |
| 784226 | 2 | yes | 7,02 | 13,69 | GH30 |
| 1401866 | 31 | yes | 6,93 | 13,40 | GH5 |
| 1063890 | 48 | no | 10,35 | 12,17 | GH5 |
| 1412845 | 373 | yes | 10,52 | 15,64 | GH5 |
| 1461559 | 372 | no | 9,37 | 15,77 | GH51 |
| 1477052 | 26 | yes | 7,09 | 15,16 | GH6 |
| 1347545 | 372 | yes | 6,11 | 15,94 | GH7 |
| 355477 | 400 | yes | 6,54 | 14,83 | GH7 |
| 1407721 | 396 | yes | 9,84 | 13,29 | GH76 |
| 397425 | 397 | yes | 7,70 | 12,62 | GH93 |
