# Supplementary material for: Integrative visual omics of the white-rot fungus Polyporus brumalis exposes the biotechnological potential of its oxidative enzymes for delignifying raw plant biomass
Source: Biotechnol Biofuels. 2018 Jul 23;11:201. doi: 10.1186/s13068-018-1198-5 (PMC6055342; doi:10.1186/s13068-018-1198-5)
Supplement: Supplementary file 7 — Additional file 7: Table S4. List of the core set of genes differentially highly transcribed at Day 4, 10 and 15 of SSF on wheat straw. [file 13068_2018_1198_MOESM7_ESM.docx]

**Table S4.** List of the core set of genes differentially highly transcribed at Day 4, 10 and 15 of SSF on wheat straw. The genes coding for P450s, GSTs, permeases and transporters which clustered into neighboring nodes of the Tatami map are highlighted in grey boxes.

|  |  | **detected in the secretome** | **Log2 normalized read counds** | | | | **CAZyme expert annotation** |  |
| --- | --- | --- | --- | --- | --- | --- | --- | --- |
| **protID** | **nodeID** |  | **control** | **Day 4** | **Day 10** | **Day 15** |  | **kog definition** |
| 784226 | 2 | yes | 7,02 | 13,69 | 12,40 | 12,83 | GH30 |  |
| 1447784 | 2 | yes | 7,62 | 14,62 | 13,73 | 14,53 | GH28 |  |
| 1404492 | 2 | yes | 7,67 | 13,56 | 13,30 | 12,93 | CE16 |  |
| 1486135 | 2 | yes | 5,39 | 14,03 | 13,48 | 13,61 | CE15 |  |
| 1456088 | 2 | yes | 7,04 | 13,93 | 14,38 | 12,62 | CE1 |  |
| 1520578 | 25 | yes | 8,24 | 11,88 | 12,39 | 12,88 | GH10 |  |
| 1349191 | 47 | yes | 9,56 | 13,41 | 12,45 | 12,90 | GH15 |  |
| 1403153 | 47 | yes | 8,87 | 13,72 | 12,95 | 13,62 | AA9 |  |
| 224000 | 209 | yes | 10,53 | 12,90 | 13,53 | 14,08 | GH3 |  |
| 615361 | 209 | yes | 10,86 | 12,82 | 13,23 | 13,43 | GH3 |  |
| 1484581 | 209 | yes | 11,18 | 13,28 | 13,18 | 13,32 | GH16 |  |
| 1399288 | 209 | yes | 11,43 | 13,63 | 13,92 | 13,34 | AA3_2 |  |
| 1347545 | 372 | yes | 6,11 | 15,94 | 15,49 | 14,02 | GH7 |  |
| 1461559 | 372 | yes | 9,37 | 15,77 | 14,86 | 14,89 | GH51 |  |
| 1339229 | 372 | yes | 7,41 | 15,00 | 14,53 | 13,60 | AA9 |  |
| 1486819 | 372 | yes | 8,52 | 16,89 | 14,66 | 12,89 | AA2 |  |
| 1412845 | 373 | yes | 10,52 | 15,64 | 13,61 | 13,96 | GH5_22 |  |
| 1498325 | 395 | yes | 9,81 | 12,50 | 14,54 | 15,83 | GH27 |  |
| 1407721 | 396 | yes | 9,84 | 13,29 | 13,20 | 12,79 | GH76 |  |
| 16190 | 397 | yes | 7,95 | 12,55 | 13,56 | 13,20 | GH10 |  |
| 1437352 | 397 | yes | 7,84 | 11,58 | 14,01 | 15,12 | GH2 |  |
| 397425 | 397 | yes | 7,70 | 12,62 | 13,80 | 13,66 | GH93 |  |
| 1395444 | 397 | yes | 8,47 | 11,36 | 13,25 | 13,59 | CE16 |  |
| 1364967 | 417 | yes | 10,76 | 17,12 | 16,14 | 15,71 | AA3_3 |  |
| 1487275 | 417 | yes | 9,84 | 18,95 | 17,82 | 15,66 | AA2 |  |
| 897918 | 418 | yes | 8,06 | 15,10 | 16,76 | 16,49 | AA2 |  |
| 1412926 | 418 | yes | 7,42 | 14,95 | 17,04 | 16,86 | AA2 |  |
| 713930 | 418 | yes | 8,38 | 17,61 | 18,31 | 17,41 | AA5 |  |
| 1557562 | 418 | yes | 6,06 | 16,87 | 17,30 | 16,83 | AA5 |  |
| 1412498 | 420 | yes | 6,99 | 12,54 | 14,36 | 15,35 | CE16 |  |
| 832761 | 420 | yes | 5,89 | 13,41 | 14,26 | 15,62 | CE12 |  |
| 1347226 | 420 | yes | 6,63 | 12,72 | 15,16 | 16,27 | AA2 |  |
| 1185543 | 420 | yes | 7,00 | 12,95 | 15,69 | 16,08 | AA2 |  |
| 1452357 | 2 |  | 6,67 | 13,42 | 12,69 | 13,11 |  | Predicted transporter (major facilitator superfamily) |
| 1398198 | 2 |  | 7,49 | 14,28 | 13,27 | 12,95 | CYP53 | Cytochrome P450 CYP3/CYP5/CYP6/CYP9 subfamilies |
| 1364780 | 2 |  | 7,80 | 13,22 | 13,27 | 13,74 | CYP5150 | Cytochrome P450 CYP3/CYP5/CYP6/CYP9 subfamilies |
| 1516684 | 47 | yes | 8,94 | 13,01 | 13,15 | 12,92 |  | Glutathione S-transferase |
| 1228724 | 47 |  | 8,50 | 13,02 | 12,99 | 12,75 | CYP512 | Cytochrome P450 CYP4/CYP19/CYP26 subfamilies |
| 1537019 | 47 |  | 8,99 | 12,94 | 13,20 | 13,65 | CYP5150 | Cytochrome P450 CYP4/CYP19/CYP26 subfamilies |
| 1410594 | 47 |  | 9,25 | 12,39 | 13,06 | 13,04 | CYP512 | Cytochrome P450 CYP3/CYP5/CYP6/CYP9 subfamilies |
| 1410802 | 47 |  | 9,59 | 12,38 | 12,51 | 12,93 |  | Predicted transporter (major facilitator superfamily) |
| 1467380 | 47 |  | 8,38 | 13,11 | 13,08 | 12,92 |  | Permease of the major facilitator superfamily |
| 1422437 | 232 |  | 11,26 | 14,10 | 12,93 | 12,57 |  | Predicted transporter (major facilitator superfamily) |
| 40616 | 232 |  | 10,41 | 13,73 | 12,62 | 12,83 |  | Predicted transporter (major facilitator superfamily) |
| 1364150 | 232 | yes | 9,81 | 13,35 | 12,80 | 12,41 |  | Glutathione S-transferase |
| 1487024 | 186 |  | 10,31 | 13,04 | 12,07 | 12,01 | CYP5144 | Cytochrome P450 CYP2 subfamily |
| 1416167 | 186 |  | 9,63 | 11,92 | 12,44 | 12,20 | CYP5144 | Cytochrome P450 CYP2 subfamily |
| 1487694 | 186 |  | 9,68 | 11,80 | 11,96 | 11,72 |  | Permease of the major facilitator superfamily |
| 1486070 | 186 |  | 10,09 | 12,27 | 11,89 | 12,04 |  | Permease of the major facilitator superfamily |
| 1334848 | 185 |  | 10,46 | 12,68 | 12,70 | 13,35 |  | Predicted transporter (major facilitator superfamily) |
| 1451844 | 209 |  | 11,09 | 12,72 | 13,25 | 12,91 |  | Predicted transporter (major facilitator superfamily) |
| 1399835 | 373 | yes | 10,78 | 15,12 | 15,21 | 15,00 |  | Glutathione S-transferase |
| 1357910 | 395 | yes | 10,55 | 14,03 | 15,89 | 16,38 |  | Glutathione S-transferase |
| 1399478 | 396 |  | 10,14 | 13,88 | 13,22 | 13,21 | CYP512 | Cytochrome P450 CYP4/CYP19/CYP26 subfamilies |
| 1361826 | 396 |  | 9,26 | 14,65 | 13,70 | 13,10 | CYP512 | Cytochrome P450 CYP3/CYP5/CYP6/CYP9 subfamilies |
| 1404557 | 396 |  | 10,31 | 13,59 | 13,12 | 13,27 | CYP5137 | Cytochrome P450 CYP4/CYP19/CYP26 subfamilies |
| 1402446 | 396 |  | 9,45 | 14,28 | 13,10 | 12,68 | CYP5136 | Cytochrome P450 CYP4/CYP19/CYP26 subfamilies |
| 540241 | 395 |  | 9,58 | 13,49 | 14,41 | 15,13 |  | Permease of the major facilitator superfamily |
| 1362876 | 396 |  | 9,86 | 14,32 | 13,31 | 13,38 |  | Predicted transporter (major facilitator superfamily) |
| 1454421 | 396 |  | 9,42 | 14,31 | 12,77 | 12,61 |  | Permease of the major facilitator superfamily |
| 334352 | 397 |  | 8,43 | 12,57 | 13,34 | 14,48 |  | Predicted transporter (major facilitator superfamily) |
| 1394716 | 397 |  | 9,03 | 11,00 | 13,86 | 14,10 |  | Permease of the major facilitator superfamily |
