# Supplementary material for: Integrative visual omics of the white-rot fungus Polyporus brumalis exposes the biotechnological potential of its oxidative enzymes for delignifying raw plant biomass
Source: Biotechnol Biofuels. 2018 Jul 23;11:201. doi: 10.1186/s13068-018-1198-5 (PMC6055342; doi:10.1186/s13068-018-1198-5)
Supplement: Supplementary file 8 — Additional file 8: Figure S3. Phylogeny of the AA3 predicted proteins from Polyporus brumalis and biochemically characterized fungal AA3s. Sequence descriptions include accession numbers from JGI Mycocosm, NBCI or PDB. The tree was constructed with a strategy similar to Sützl et al. (Sützl et al. 2018 [22]). Sequences were aligned using M-coffee (Wallace et al. [69]) with default settings. Phylogeny was inferred using PhyML (Guindon et al. [70]) and WAG amino acid substitution model (Whelan and Goldman [71]). Branch support was calculated by 500 bootstrap repetitions. The tree was visualized in iTOL (Letunic and Bork [72]). [file 13068_2018_1198_MOESM8_ESM.pptx]

## Slide 1
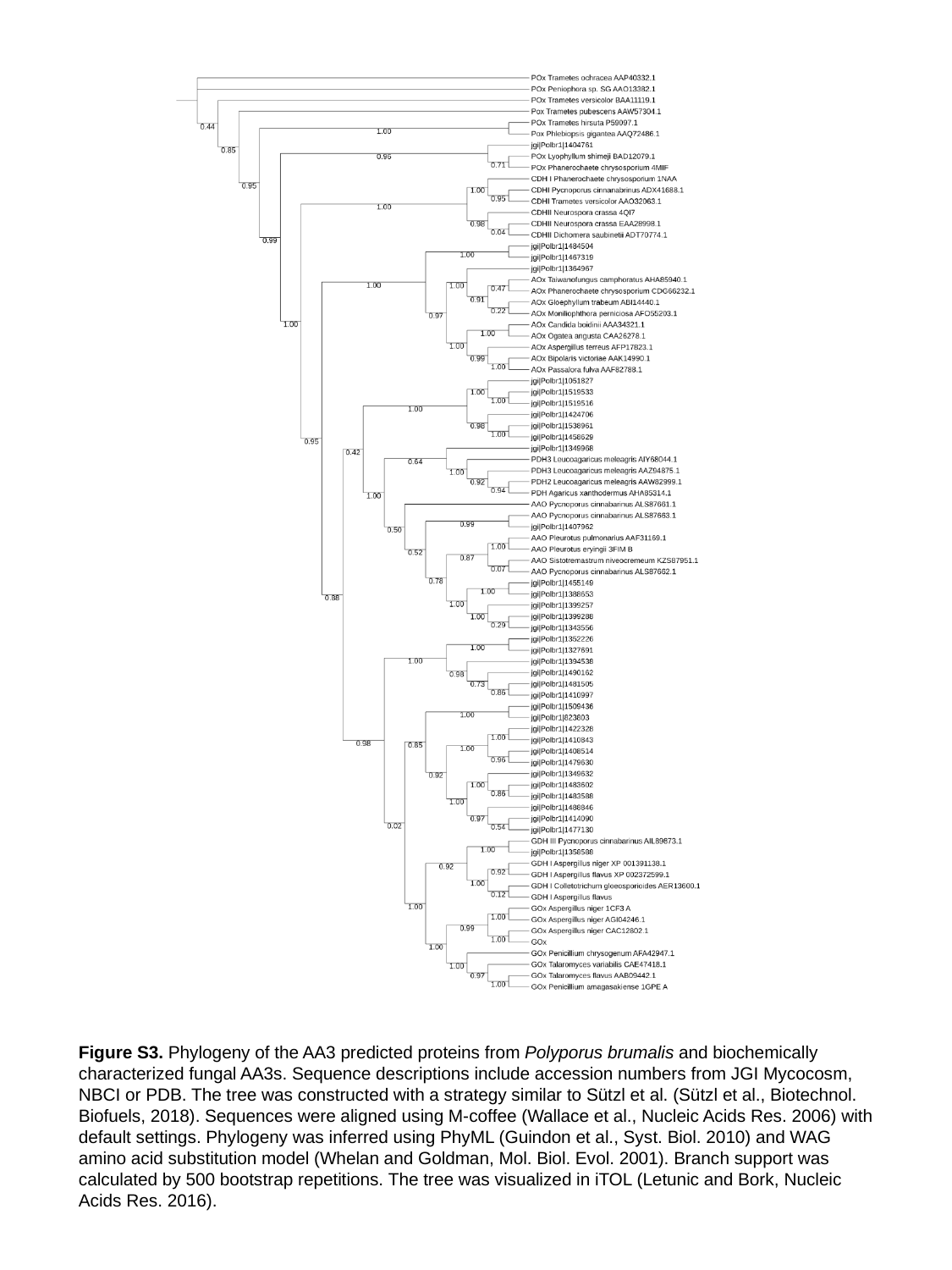

Figure S3. Phylogeny of the AA3 predicted proteins from Polyporus brumalis and biochemically characterized fungal AA3s. Sequence descriptions include accession numbers from JGI Mycocosm, NBCI or PDB. The tree was constructed with a strategy similar to Sützl et al. (Sützl et al., Biotechnol. Biofuels, 2018). Sequences were aligned using M-coffee (Wallace et al., Nucleic Acids Res. 2006) with default settings. Phylogeny was inferred using PhyML (Guindon et al., Syst. Biol. 2010) and WAG amino acid substitution model (Whelan and Goldman, Mol. Biol. Evol. 2001). Branch support was calculated by 500 bootstrap repetitions. The tree was visualized in iTOL (Letunic and Bork, Nucleic Acids Res. 2016).
